# Endoscopic diagnosis and treatment of superficial non-ampullary duodenal epithelial tumors: A review

**DOI:** 10.2478/jtim-2023-0102

**Published:** 2023-09-02

**Authors:** Zheng Zhao, Yue Jiao, Shuyue Yang, Anni Zhou, Guiping Zhao, Shuilong Guo, Peng Li, Shutian Zhang

**Affiliations:** Department of Gastroenterology, Beijing Friendship Hospital, Capital Medical University, Beijing 100050, China

**Keywords:** superficial non-ampullary duodenal epithelial tumors, endoscopic diagnosis, endoscopic treatment

## Abstract

The surface of the small bowel mucosa is covered more than any other section of the digestive canal; however, the overall prevalence of small bowel tumors of the whole gastrointestinal tract is evidently low. Owing to the improvement in endoscopic techniques, the prevalence of small bowel tumors has increased across multiple countries, which is mainly due to an increase in duodenal tumors. Superficial non-ampullary duodenal epithelial tumors (SNADETs) are defined as tumors originating from the non-ampullary region in the duodenum that share similarities and discrepancies with their gastric and colorectal counterparts in the pathogenesis and clinicopathologic characteristics. To date, white light endoscopy (WLE) remains the cornerstone of endoscopic diagnosis for SNADETs. Besides, narrow-band imaging (NBI) techniques and magnifying endoscopy (ME) have been widely used in the clinic and endorsed by multiple guidelines and consensuses for SNADETs’ evaluation. Confocal laser endomicroscopy (CLE), endocytoscopy (ECS), and artificial intelligence (AI) are also up-and-coming methods, showing an exceptional value in the diagnosis of SNADETs. Similar to the endoscopic treatment for colorectal polyps, the choices for SNADETs mainly include cold snare polypectomy (CSP), endoscopic mucosal resection (EMR), endoscopic submucosal dissection (ESD), and laparoscopic endoscopic cooperative surgery (LECS). However, owing to the narrow lumen, rich vascularity, weak muscle layer, abundant Brunner’s gland, and the hardship of endoscope control, the duodenum ranks as one of the most dangerous operating areas in the digestive tract. Therefore, endoscopists must anticipate the difficulties in endoscopic maneuverability, remain aware of the increased risk of complications, and then select the appropriate treatment according to the advantages and disadvantages of each method.

## Introduction

In general, superficial non-ampullary duodenal epithelial tumors (SNADETs) are rarer than other gastrointestinal (GI) tumors; however, studies performed in recent years have reported an increase in the incidence of these lesions.^[1]^ Notably, endoscopy has adopted an irreplaceable role in the stepwise understanding of SNADETs. With the improved availability of endoscopic screening and advances in endoscopic techniques,^[2]^ a broader investigation of the diagnosis and treatment of SNADETs has emerged.^[3,4]^ Recently, the European Society of Gastrointestinal Endoscopy (ESGE) issued the very first clinical practice guideline on SNADETs.^[5]^ Nevertheless, current diagnostic and therapeutic strategies still have room for improvement. This review seeks to shed light on the current status of endoscopic modalities for the diagnosis and treatment of SNADETs and discuss the remaining challenges.

## Clinical features of SNADETs

SNADETs are defined as tumors originating from the non-ampullary region in the duodenum, consisting of dysplastic glandular epithelium, which may show intestinal-type or pyloric gland differentiation (also called gastric-type).^[6]^ In epidemiology, the estimated prevalence of non-ampullary duodenal adenoma is 0.03%–0.40%,^[7, 8, 9]^ and the incidence of non-ampullary duodenal cancer is 23.7 per 1,000,000 person-years in the up-to-date Japanese survey study.^[10]^ Most of the lesions are detected incidentally by endoscopic examinations as they are usually small and asymptomatic. However, large lesions can cause obstructive symptoms or complaints related to tumor progression and metastasis.^[11]^ Only very few cases have been reported from the onset of metastatic symptoms.^[12]^ SNADETs are less often sporadic than their ampullary counterparts, as approximately 60% of SNADETs occur in patients with familial adenomatous polyposis (FAP).^[13]^ Besides, other predisposing genetic syndromes, including MUTYH-associated polyposis (MAP), Lynch syndrome (LS), and Peutz–Jeghers syndrome (PJS), are associated with an increased risk of SNADETs.^[14, 15, 16]^ Therefore, when SNADET is detected in a patient, an extra colonoscopy is strongly recommended.^[17]^ Further, specific management strategies for these polyposis syndromes are discussed in another guideline from ESGE.^[18]^

**Figure 1:**
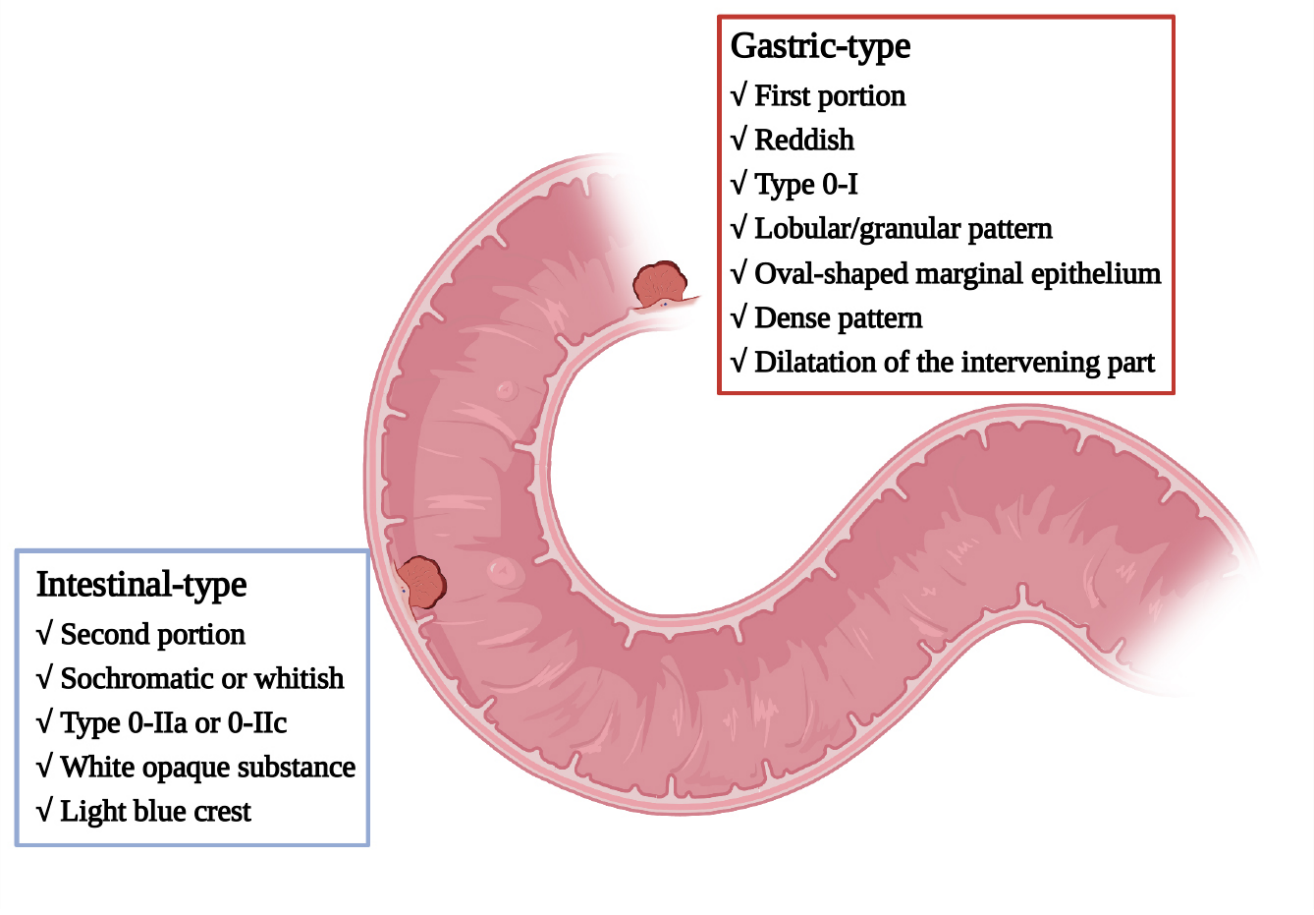
Summary of the clinicopathologic characteristics of gastric-type and intestinal-type SNADETs. SNADETs: superficial non-ampullary duodenal epithelial tumors.

The revised Vienna classification (VCL) (Table 1) is now a well-established clinicopathologic parameter for evaluating the malignant levels of SNADETs. This parameter generally divides the lesions into mucosal low-grade adenoma (VCL 3) and mucosal high-grade adenoma/carcinoma (VCL 4/5).^[19]^ Most of the lesions are found to be VCL 3 at the initial diagnosis, and some could progress to VCL 4/5.^[20]^ As mentioned above, SNADETs can be further subdivided into intestinal-type and gastric-type based on immunohistochemical (IHC) staining: CDX2 and MUC2 for intestinal-type and MUC5AC and MUC6 for gastric-type.^[21,22]^ Accordingly, the pathogenesis from adenoma to adenocarcinoma differs between intestinal-type and gastric-type.^[23]^ Similar to colorectal cancer (CRC), the adenoma– carcinoma sequence model involves tumor progression in SNADETs of intestinal-type. In contrast, the gastric-type seems to progress *de novo*, which is associated with the Wnt/β-catenin pathway. Many studies have confirmed the differences in the clinicopathologic characteristics between the two types of SNADETs,^[24, 25, 26]^ as shown in Figure 1.

**Table 1:** The revised Vienna classification of gastrointestinal epithelial neoplasia

**Category**	**Diagnosis**
1	Negative for neoplasia
2	Indefinite for neoplasia
3	Mucosal low-grade neoplasia
	Low-grade adenoma
	Low-grade dysplasia
4	Mucosal high-grade neoplasia
4.1	High-grade adenoma/dysplasia
4.2	Noninvasive carcinoma (carcinoma in situ)
4.3	Suspicious for invasive carcinoma
4.4	Intramucosal carcinoma
5	Submucosal invasion by carcinoma

## Endoscopic diagnosis for SNADETs

### White light endoscopy and biopsy

White light endoscopy (WLE) and biopsy are the most fundamental and essential means of diagnosing SNADETs.^[27]^ In WLE, the Paris endoscopic classification is widely used to describe the macroscopic types of SNADETs, namely, pedunculated (0-Ip), sessile (0-Is), slightly elevated (0-IIa), flat (0-IIb), slightly depressed (0-IIc), and excavated (0-III).^[28]^ According to several studies, size, color, macroscopic type, and biopsy results are important assessment indicators for endoscopic diagnosis. For example, a surveillance study (46 lesions followed up for ≥ 6 months) reported that a lesion with a diameter ≥ 20 mm and its diagnosis as high-grade dysplasia (HGD) at the first biopsy are two key indicators of a higher risk of progression to adenocarcinoma.^[20]^ In another multicenter case series involving 396 SNADETs, HGD and superficial adenocarcinoma (SAC; including carcinoma *in situ* and invasive carcinomas) were recognized to more likely present with a dimeter > 5 mm and red color.^[29]^ Furthermore, Kakushima *et al*.^[30]^ proposed a simple endoscopic scoring system to differentiate between VCL 3 and VCL 4 or higher SNADETs (Table 2), where a score ≥ 3 points indicated the histology of VCL 4 or higher, with sensitivity and specificity of 88% and 86%, respectively. In the follow-up study, the researchers mentioned extra endoscopic features of submucosal invasion under WLE, including submucosal tumor-like appearance (defined as a smooth or gentle elevation at the margin of a lesion) and location at the oral side of the ampulla, even if the lesion is ≤ 10 mm.^[31]^ Overall, WLE remains the cornerstone of endoscopic diagnosis for SNADETs, as it is still a fundamental skill for every endoscopist. However, biopsy seems to be less recommended as it can affect the visual observation and the sequential treatment procedure.^[32]^ Besides, Kinoshita *et al*.^[33]^ revealed that the accuracy of SNADETs biopsy was unsatisfactory, with a sensitivity of 37.5% and specificity of 83.1%. Tsuji *et al*.^[34]^ also showed that the sensitivity and specificity were 89% and 14%, respectively, and that optical findings would be obstructed by the preceding biopsy injuries. Additionally, both studies emphasized that the unexpected fibrosis after biopsy might increase the difficulty in treatment, with predetermined endoscopic mucosal resection (EMR) being converted to endoscopic submucosal dissection (ESD). Collectively, WLE and biopsy play significant roles in the endoscopic diagnosis of SNADETs owing to their convenience and feasibility during endoscopic screen. Nonetheless, both WLE and biopsy require plenty of experience to avoid misdiagnosis, especially the biopsy procedure. Endoscopists must have considerable expertise to handle an exact specimen with minimal interference in the subsequent endoscopic treatment. Given the inadequacy, more novel tools have been introduced to complement the existing methods, and these techniques will be discussed in the next part.

**Table 2:** A simple endoscopic scoring system for SNADETs^[30]^

Endoscopic finding		Score	
	**0**	**1**	**2**
Lesion diameter	< 10 mm	≥ 10 mm	
Color	White	Isochromatic	Red
Macroscopic type	Is, Ip, IIa without depression	Any type with depression or mixed type	
Nodularity	Uniform	Heterogeneous or none	

SNADETs: superficial non-ampullary duodenal epithelial tumors.

### Narrow band imaging and magnifying endoscopy

Narrow-band imaging (NBI) techniques and magnifying endoscopy (ME) have been widely used in the clinic, as endorsed by multiple guidelines and consensuses for diagnosing GI tumors.^[35,36]^ For SNADETs, NBI and ME have shown unique value in differentiating neoplastic lesions from non-neoplastic lesions, acting as an important complementary tool to WLE.^[37,38]^ Many researchers have proposed their original diagnostic algorithms for SNADETs using ME-NBI, which achieved excellent performances for distinguishing VCL 4/5 lesions from VCL 3 lesions.^[39, 40, 41]^ In particular, the absence of white opaque substance (WOS) and lack of milk-white mucosa (MWM) findings were significantly associated with VCL 4/5 lesions.^[42,43]^ Based on the WLE scoring system, Ishii *et al*.^[44]^ developed another scoring system using ME- NBI. Besides the diameter and color of the lesion, they included the surface pattern and vessel pattern (observed by ME-NBI) into the scoring system, as shown in Table 3, where a score ≥ 3 served as the cut-off for VCL 4/5 lesions, with a sensitivity and specificity of 95% and 93%, respectively. The application of ME-NBI opens a chapter in the endoscopic diagnosis for SNADETs. Additionally, chromoendoscopy was introduced as an adjuvant tool to provide more details of the lesion with “a colorful perspective,” which ultimately has become a valuable aid to WLE and ME-NBI.^[45]^ Crystal violet (CV) staining, accompanied with ME-NBI, has been confirmed to be the most suitable choice for SNADETs.^[46]^ For instance, Toya *et al*.^[47]^ designed a diagnostic algorithm of ME-NBI with CV staining to distinguish VCL 4/5 from VCL 3. Briefly, they subdivided the surface patterns into four categories (a convoluted pattern, a leaf-like pattern, a reticular/sulciolar pattern, a pinecone pattern) and organized two structure patterns (regular pattern and irregular pattern). In their theory, VCL 4/5 lesions presented with multiple surface patterns or irregular structure patterns, while VCL 3 lesions were more likely to manifest single surface patterns and regular structure patterns. Regretfully, due to the lack of larger-scale analysis, more investigations are required to demonstrate the diagnostic value of this algorithm.

**Table 3:** Endoscopic findings included in the scoring system^[44]^

**Variables**	**0**	**1**	**2**
Diameter	< 10 mm	10–<20 mm	≥ 20 mm
Color	White/isochromatic	Red	
Surface pattern	Regular	Irregular	
Vessel pattern	Regular	Irregular	

### Confocal laser endomicroscopy, endocytoscopy, and artificial intelligence

Confocal laser endomicroscopy (CLE) and endocytoscopy (ECS) are novel endoscopic techniques that provide an unprecedented high-resolution assessment of GI mucosal histology at the cellular and sub-cellular level, offering high hopes of achieving “optical biopsies” of nearly any accessible endoluminal surface.^[48,49]^ To date, two types of CLE have been introduced, endoscopic-based CLE (eCLE) and probe-based CLE (pCLE), with pCLE appearing more practical and helpful in the duodenum.^[50]^ Shahid *et al*.^[51]^ elucidated the high diagnostic efficacy of pCLE in both *ex vivo* pathology and *in vivo* duodenal polyps. Likewise, Tahara *et al*.^[52]^ indicated that the dark epithelium and distorted crypt structure are characteristic pCLE features of neoplasia and cancer in SNADETs, with a sensitivity and specificity of nearly 100%. In terms of ECS, Hirose *et al*.^[53]^ illustrated that ECS diagnosis with methylene blue staining could achieve a high accuracy to predict the histology of SNADETs. Moreover, Muramoto *et al*.^[54]^ created an original ECS classification for the diagnosis of SNADETs based on cell nuclear morphology, which showed significantly superior diagnostic effects compared to preoperative biopsy. Nevertheless, with the requirements of expensive equipment and extra proficiency, gaining popularity at all levels of the health sector is not an easy task, thereby immensely constraining the scale-up. Beyond the exciting updates in facilities, artificial intelligence (AI) has gradually become a rising star in the field of endoscopic diagnosis.^[55]^ Inoue *et al*.^[56]^ made the first attempt to apply convolutional neural networks to the detection of SNADETs in endoscopic images, which required 12 s to identify the lesions in 399 photos, with an accuracy of 94.7%. Besides various electronic and information technologies, the biological molecule is becoming an integral part of research on many other GI diseases.^[57, 58, 59]^ Accordingly, we believe that it will also optimize the diagnosis of SNADETs. With the rapid advances in both “hardware” and “software,” the technological revolution will contribute to the increasing awareness and recognition of SNADETs.

## Endoscopic treatment for SNADETs

Similar to the endoscopic treatment for colorectal polyps, the choices for SNADETs mainly include cold snare polypectomy (CSP), EMR, underwater EMR (UEMR), ESD, and laparoscopic endoscopic cooperative surgery (LECS).^[60, 61, 62]^ However, characterized by a narrow lumen, rich vascularity, weak muscle layer, abundant Brunner’s gland, and the hardship of endoscope control, the duodenum ranks as one of the most dangerous operating areas in the digestive tract.^[63]^ Furthermore, unlike the intensive experience in colonoscopy,^[64]^ the experience of endoscopic treatment in the duodenum remains limited. Therefore, endoscopists must anticipate the difficulties in endoscopic maneuverability, be aware of the increased risk of complications, and select the appropriate treatment according to the advantages and disadvantages of each method (Table 4).

**Table 4:** Characteristics and indications of endoscopic treatments

**Treatment methods**	**Advantages**	**Disadvantages**	**Recommended indications**
CSP	Simple, safe, low incidence of complications	Incomplete resection and local recurrence	Lesion size < 6 mm, especially suitable for FAP
EMR (UEMR)	Convenient, relatively low incidence of complications	Relatively low en bloc resection rate	Lesion size ≤ 20 mm
ESD	High en bloc resection rate irrespective of lesion size	High incidence of complications and proficiency requirement	Lesion size > 20 mm
LECS	Less invasive compared to pancreaticoduodenectomy and secure defect closure	Postoperative obstruction and not suitable for lesions adjacent to ampulla	Lesion size > 20 mm and endoscopically unresectable

CSP: cold snare polypectomy; EMR: endoscopic mucosal resection; ESD: endoscopic submucosal dissection; FAP: familial adenomatous polyposis; LECS: laparoscopic endoscopic cooperative surgery; UEMR: underwater EMR.

### EMR and UEMR

EMR is a well-established method for removing GI tumors. According to the ESGE guidelines, EMR is the first choice of endoscopic resection for SNADETs.^[5]^ Generally, the procedure refers to the isolation of the lesion via a submucosal fluid injection and snare excision of the isolated dysplastic lesion. Basically, lesions ≤20 mm can be removed *en bloc* by EMR, and most endoscopists can perform this procedure.^[65]^ The high rates of complete resection (range 90%–100 %) were confirmed by multiple studies.^[66, 67, 68, 69, 70]^ Despite simple manipulation, the complications cannot be completely avoided. Given the difficulties of endoscopic therapy in the duodenum, several additional studies revealed higher complications rates than lesions elsewhere in the digestive tract, as the morbidities of bleeding and perforation ranged 0–22.2% and 0–4.8%, respectively.^[71, 72, 73, 74]^ Moreover, a recent study showed that *en bloc* resection was almost impossible for lesions ≥30 mm.^[75]^ Dealing with these giant lesions, the operators may perform piecemeal resection in EMR procedures. With the requirement for piecemeal resection, larger lesions often result in a higher risk of local recurrence (range 6%–23%). In fact, the highest incidence of recurrence was 37% in a retrospective study.^[70,76-78]^ Nonetheless, EMR retains an important role in endoscopic resection, regardless of its shortcomings. Moreover, endoscopists have identified various measures to improve this technique. UEMR counts as a reliable advanced technique that originated from conventional EMR. UEMR was first introduced by Binmoeller *et al*.^[79]^ for managing SNADETs. In conventional EMR, the lumen is permanently dilated by air, causing the duodenum wall to be thinner and particularly vulnerable to collision damage. In UEMR, filling with water can retain the thickness and configuration of the duodenum, which helps to reduce the thermal injury. Of note, the water pressure also helps terminate bleeding. Besides, water can eliminate the need for submucosal injection, with a “floating” effect on the mucosa and submucosa.^[80, 81, 82]^ For instance, Kiguchi *et al*.^[83]^ compared the clinical outcomes between UEMR and conventional EMR and found that UEMR could notably assist in the avoidance of ESD conversion. Notably, no significant differences were observed between the complication rates of the two groups. Nevertheless, UEMR exhibited a relatively low R0 resection rate and *en bloc* resection rate than EMR. Altogether, the outcome of conventional EMR might be overestimated as the difficult cases were excluded because of the conversion to ESD in the study. Besides, the researchers suggested that devices dedicated to UEMR procedures should be developed. In a follow-up study, the researchers demonstrated the feasibility of partial submucosal injection technique combining UEMR (PI-UEMR) for SNADETs.^[84]^ To remove the lesion with non-lifting sign during UEMR, they administered a submucosal injection on the difficult side (mostly anal side) of the lesion and then resected the lesion by recognizing a sufficient margin. In 30 patients who underwent PI-UEMR, only one case of immediate bleeding occurred without any other delayed complications. In addition, UEMR was shown to be appropriate for treating residual lesions after previous endoscopic resection.^[85,86]^ Altogether, EMR and the derived techniques (like UEMR and PI-UEMR) are top selections, while ESD is more suitable for large and complex lesions, as discussed in the next section.

### Endoscopic submucosal dissection

ESD for SNADETs is more complex than for lesions from other locations along the GI tract. In fact, the ESGE guidelines suggest ESD should be considered for exclusive indications only in the hands of an expert.^[5]^ Usually, ESD is the first strategy of choice for lesions > 20 mm.^[60]^ Besides, lesions presenting non-lifting conditions are always eventually removed by ESD, despite the initial procedure.^[87]^ However, ESD is regarded as a “double-edged sword” due to its reliable removal efficacy and high incidence of complications, even in experienced endoscopy units.^[88,89]^ In terms of the advantages, duodenal ESD was proved to achieve an *en bloc* resection rate greater than 90 %, even for lesions larger than 20 mm.^[90,91]^ Moreover, multiple comparative studies showed that duodenal ESD could reach higher R0 resection rates for giant lesions, with no significant differences found in long-term outcomes between ESD and EMR.^[91, 92, 93]^ Nevertheless, the higher risk of procedure-related complications is the limiting factor. As observed in multiple studies, the bleeding rate increases up to 46% and the perforation rate ranges from 13 % to 50 %. Even more, 17% of the cases accepted additional surgery in a study.^[88,90,94–96]^ The exact reasons can be summarized as follows: (1) the narrow lumen of the duodenum restricts the reverse method of manipulation; (2) deep intubation into the duodenum shortens the operable parts for endoscopists, causing great difficulty in operational stability; (3) the C-loop structure of the duodenum makes it easy to slip off the target; (4) abundant Brunner’s gland impairs the effectiveness of submucosal injection; (5) rich blood supply and active intestinal peristalsis obstruct the dissection; (6) poor expandability of the duodenal mucosa makes it difficult to close the defect; (7) the thin muscularis propria and muscularis mucosa are easily damaged and induce perforation; and (8) the transfer to surgery faces extra challenges. Therefore, the discovery of effective endoscopic methods is markedly desired, and many researchers have made meaningful attempts to attenuate these complications. For example, covering the wound with polyglycolic acid (PGA) sheets helped to prevent delayed perforation after duodenal ESD.^[97,98]^ Some endoscopists also suggest indwelling endoscopic nasobiliary and pancreatic duct drainage (ENBPD) tubes for incomplete closed lesions;^[99]^ this is because ENBPD is thought to be a helpful prophylaxis for protecting the duodenal mucosa from the erosion of bile and pancreatic juice.^[100]^ Bleeding is another main complication of duodenal ESD, which appears even more commonly than perforation. Overall, guaranteeing closure of the mucosal defects is the essential solution.^[101]^ Typically, the application of endoscopic clipping is the most convenient and primary method of preventing bleeding after duodenal ESD.^[102]^ Recently, an over-the-scope clip (OTSC) system was reported to help close the defect and reduce delayed bleeding after duodenal ESD.^[103,104]^ Subtly, the coordination of endoscopic clipping and suturing has become a highlight, which fully unfolded the craft and creativity of endoscopists.^[105, 106, 107]^ Remarkable efforts have also been made to improve endoscopic instruments for cutting, with an aim to decrease electrical injury in ESD procedures. Hook knife and scissors-type knife (clutch cutter) have been acknowledged by many endoscopists as practicable and safe equipment in duodenal ESD.^[108, 109, 110, 111]^ Thoughtfully, ensuring maneuverability in the confined room has been an issue for endoscopists. The double-balloon endoscope has been described as a helpful tool to stabilize the operation of the endoscope tip, which can be especially suitable for duodenal ESD.^[112]^ Many researchers have also emphasized the value of various types of traction techniques in duodenal ESD. Goda *et al*.^[113]^ revealed the efficacy of ring-shaped thread counter traction, which could provide sufficient operation view in duodenal ESD. Tashima *et al*.^[114]^ performed traction-assisted ESD with dental floss and a clip for a lesion with severe fibrosis and used multiple clip-and-thread traction to remove a large lesion located in the duodenal bulb.^[115]^ With a better operation view, the rate of bleeding and perforation can be effectively reduced. In summary, the better safety and more effortless procedure of EMR lead to a higher priority for removing SNADETs. Furthermore, ESD remains of vital indispensability for large and complex lesions; however, endoscopists must be aware of procedure-related complications.

### CSP and LECS

Compared to EMR and ESD, CSP and LECS are less popular; however, both can be exploited in some special situations to gain desirable effects. CSP is a physical method that uses a snare without an electrical current, ultimately reducing the potential injury caused by electrically induced heat. Relatively, the solo physical force of the snare limits the removal efficacy. Thus, the ESGE guidelines suggest that CSP is only suitable for small and nonmalignant SNADETs (<6 mm in size).^[5]^ In particular, CSP is appropriate for FAP patients with numerous and small duodenal polyps, owing to its simplicity and safety.^[116, 117, 118]^ Nevertheless, the relatively high incomplete resection rate is an obvious shortcoming of CSP, leading to its limited application in SNADETs.^[119,120]^ LECS is a creative approach first introduced by Hiki *et al*.^[121]^ for GI stromal tumor dissection. Technically, the procedure involves laparoscopic resection with endoscopic guidance and endoscopic resection with surgical repair.^[122,123]^ In theory, LECS should fulfill both fewer complications related to endoscopic resection and slighter damage than traditional surgery.^[124,125]^ LECS requires high-level cooperation between endoscopists and surgeons to achieve safe management of SNADETs, which virtually raises the threshold for medical facilities. Although LECS has been reported to exhibit an exemplary safety and efficacy for reducing adverse events and recurrence,^[123,126]^ more extensive prospective studies are still needed to prove these conclusions.

## Conclusions and outlook

Currently, endoscopic management of GI tumors is advancing with remarkable momentum. Due to the rarity, SNADETs, especially sporadic cases, still lack standardized diagnostic criteria and treatment approaches. To improve diagnostic levels, internationally recognized criteria should be established according to the advances in optical techniques. As mentioned above, multiple choices are available for treating SNADETs; yet the final decision is frequently based on the endoscopist’s personal experience. Further, the complications related to the endoscopic procedure remain as challenges. Therefore, more consensus guidelines are urgently needed to normalize the workflow of the endoscopic treatment for SNADETs. As it stands, EMR can be first considered for the endoscopic resection of SNADETs. For larger lesions, especially those with the possibility of deep invasion, ESD and LECS are more suitable after detailed and rigorous preoperative assessment of efficacy and safety. As presented in this review, many leading-edge methods have been applied practically to prevent complications. Ultimately, the endoscopists must carefully manage SNADETs and conduct more extensive investigations to determine whether the technique will help improve outcomes.
